# Commentary: ZIC2 drives colorectal cancer progression by regulating QPRT-mediated cell migration

**DOI:** 10.3389/fimmu.2026.1823749

**Published:** 2026-04-13

**Authors:** Keying Wang, Jie Lin

**Affiliations:** Shenzhen Hospital (Futian) of Guangzhou University of Chinese Medicine, Shenzhen, Guangdong, China

**Keywords:** colorectal cancer (CRC), metabolic reprogramming, QPRT, therapeutic potential, tumor microenvironment, ZIC2

## Introduction

The relentless pursuit of novel biomarkers and therapeutic targets in colorectal cancer (CRC) remains a cornerstone of oncology research, driven by the disease’s persistent high mortality and the limitations of current staging systems in capturing its profound heterogeneity. In this context, the study by Zheng and colleagues provides a timely and multi-faceted investigation into the oncogenic role of Zinc Finger Protein ZIC2 in CRC (1), positioning it as a promising prognostic indicator and a regulator of cell migration through the metabolic enzyme Quinolinate Phosphoribosyltransferase (QPRT) (2). The authors adeptly employ a comprehensive strategy, weaving together pan-cancer bioinformatics, multi-omics dissection of the CRC microenvironment, and functional *in vitro* validation to build a compelling narrative. This commentary seeks to engage with the study’s significant strengths while constructively examining key mechanistic and translational questions that emerge from its findings, aiming to highlight pathways for future research that could further solidify the clinical relevance of the ZIC2-QPRT axis.

## Evaluation of mechanistic insights and methodological scope

The study’s primary strength lies in its systematic approach, establishing a clear association between high ZIC2 expression, poor patient prognosis, and aggressive cellular phenotypes. The identification of QPRT as a key downstream correlate … is a notable finding, as it connects a developmental transcription factor to a metabolic node in the kynurenine pathway (KP) (3) – a major route of tryptophan metabolism with well-established roles in immune regulation and cancer progression (4). The functional rescue experiment using phthalic acid, a QPRT inhibitor, to partially reverse ZIC2-induced migration offers direct experimental support for this regulatory relationship. However, this core mechanistic claim invites deeper scrutiny. While the dual-luciferase assay suggests ZIC2 can activate the QPRT promoter, the precise transcriptional regulatory mechanism remains to be fully elucidated. However, the pathway from promoter binding to functional outcome remains incompletely mapped.It is unclear how the transcriptional upregulation of QPRT leads to increased enzymatic activity, and more importantly, how the consequent metabolic rewiring—specifically, the elevated NAD+ biosynthesis—precisely fuels the migratory phenotype. Given the pivotal role of NAD+ in cellular bioenergetics, stress response, and its emerging function in modulating the tumor immune microenvironment, elucidating this link is of paramount importance (5). The partial rescue by phthalic acid intriguingly suggests that ZIC2’s pro-migratory effect is not exclusively dependent on QPRT, hinting at the involvement of parallel or complementary pathways. This presents a critical area for expansion; subsequent research could employ techniques like chromatin immunoprecipitation sequencing (ChIP-seq) to define the genome-wide binding landscape of ZIC2 in CRC cells and metabolomic profiling to quantify the flux through the QPRT-dependent NAD+ synthesis pathway upon ZIC2 manipulation.

From a methodological perspective, the reliance on a single CRC cell line (HCT116) for all functional validations, while common, represents a limitation that tempers the generalizability of the conclusions. CRC is characterized by distinct molecular subtypes (e.g., CMS classification) with varying pathway activations and clinical behaviors. Validating the ZIC2-QPRT axis across cell lines representing different CRC subtypes (such as SW480 for mesenchymal-like phenotypes or DLD-1 for metabolic profiles) would significantly strengthen the evidence for its broad relevance. Furthermore, this limitation transcends methodological generalization and touches upon the core of translational relevance. Colorectal cancer encompasses consensus molecular subtypes (CMS) with distinct biological behaviors and therapeutic vulnerabilities. It would be therefore critical to investigate whether the ZIC2-QPRT axis is a ubiquitous driver or a context-dependent vulnerability, predominantly active in, for instance, the metabolically dysregulated CMS3 subtype. Determining its subtype-specificity would significantly refine its potential as a therapeutic target and guide patient stratification in future clinical explorations. Besides, the compelling *in vitro* data necessitate *in vivo* corroboration. The absence of animal model data leaves an important gap in understanding the role of this axis within the complex physiology of a living tumor, including its effects on primary tumor growth, metastasis, and interaction with the immune stroma. Future studies employing orthotopic or metastatic mouse models with modulated ZIC2 expression would be invaluable in translating this cellular mechanism into a potential therapeutic context.

## Discussion and future directions

The study successfully reframes ZIC2 from a developmental factor to a credible oncogenic driver and prognostic biomarker in CRC. Its integration of spatial transcriptomics to localize ZIC2 expression predominantly within tumor cells is a particularly elegant touch, aligning the molecular finding with histological context. This correlation is particularly noteworthy as it positions ZIC2 not merely as a standalone prognostic marker, but as a potential modulator of the tumor-immune interface. A critical, unexplored question arising from this finding is whether high ZIC2 expression merely correlates with a hypermutated, immunogenic context, or whether it functionally contributes to shaping an immunosuppressive microenvironment, potentially through modulating the kynurenine pathway and its immunoregulatory metabolites (4). Future research should prioritize dissecting the causal relationship, if any, between ZIC2 and the efficacy of immunotherapy in CRC models, which would dramatically elevate the translational impact of the original study’s bioinformatic observation. The critical discussion point revolves around the chain of causality. The presented data robustly support a model where ZIC2 enhances QPRT expression, which in turn facilitates cell migration. To elevate this model, future work should aim to dissect this axis with greater mechanistic resolution. Key unresolved questions thus emerge: Does ZIC2 directly and solely transactivate the QPRT gene, or are intermediary co-factors required? Furthermore, does the consequent increase in NAD+ production directly fuel migration by regulating cytoskeletal dynamics, or does it primarily support other pro-tumorigenic processes such as oxidative stress management? Addressing these questions will clarify the most effective therapeutic node within this axis. Direct targeting of the transcription factor ZIC2 is notoriously challenging. Therefore, discerning whether the more druggable QPRT enzyme or its key downstream metabolic effectors present superior targets is crucial for translational development. A pragmatic translational roadmap could involve: first, validating QPRT as a more druggable nodal point than ZIC2 using genetic and pharmacological tools across models; second, identifying the key NAD+-consuming enzymes (e.g., PARPs, sirtuins) that execute the pro-migratory signal; and finally, exploring synergistic effects of QPRT inhibition with standard therapies or immunomodulators, leveraging the intriguing link between ZIC2 and MSI/TMB.

Furthermore, the translational potential of targeting the ZIC2-QPRT axis requires careful consideration of its expression profile. The original study indicates that ZIC2 is also expressed in tumor-infiltrating immune cells, such as monocytes/macrophages and T cells. Therefore, therapeutic strategies designed to inhibit ZIC2 in cancer cells may concurrently affect its function in these immune populations. This potential on-target/off-tumor effect could inadvertently modulate anti-tumor immunity, necessitating future research to delineate the cell type-specific roles of ZIC2 within the tumor microenvironment to inform the development of more precise targeting approaches.

In conclusion, Zheng et al. have made a substantial contribution by rigorously characterizing ZIC2’s role in CRC and proposing a novel link to QPRT-mediated metabolism. The work’s value is in establishing a strong foundational hypothesis. The logical next steps involve mechanistic deepening through multi-omics integration, validation across diverse experimental models, and exploration of the therapeutic implications of modulating this pathway, especially in the context of immunotherapy. This is particularly relevant as NAD+ metabolic enzymes, including those in salvage and biosynthesis pathways, are increasingly recognized to play dual roles in cancer cell intrinsic metabolism and in shaping the immunosuppressive tumor microenvironment (5). By pursuing these directions, the potential of ZIC2 and QPRT as biomarkers or targets can be more accurately gauged, potentially offering new strategies to intervene in colorectal cancer progression.
